# Structural Relationship of the Lipid A Acyl Groups to Activation of Murine Toll-Like Receptor 4 by Lipopolysaccharides from Pathogenic Strains of *Burkholderia mallei*, *Acinetobacter baumannii*, and *Pseudomonas aeruginosa*

**DOI:** 10.3389/fimmu.2015.00595

**Published:** 2015-11-23

**Authors:** Kirill V. Korneev, Nikolay P. Arbatsky, Antonio Molinaro, Angelo Palmigiano, Rima Z. Shaikhutdinova, Mikhail M. Shneider, Gerald B. Pier, Anna N. Kondakova, Ekaterina N. Sviriaeva, Luisa Sturiale, Domenico Garozzo, Andrey A. Kruglov, Sergei A. Nedospasov, Marina S. Drutskaya, Yuriy A. Knirel, Dmitry V. Kuprash

**Affiliations:** ^1^Engelhardt Institute of Molecular Biology, Russian Academy of Sciences, Moscow, Russia; ^2^Biological Faculty, Lomonosov Moscow State University, Moscow, Russia; ^3^Zelinsky Institute of Organic Chemistry, Russian Academy of Sciences, Moscow, Russia; ^4^Department of Chemical Sciences, Università di Napoli Federico II, Naples, Italy; ^5^CNR Institute for Polymers Composites and Biomaterials, Catania, Italy; ^6^State Research Centre of Applied Microbiology and Biotechnology, Obolensk, Russia; ^7^Shemyakin and Ovchinnikov Institute of Bioorganic Chemistry, Russian Academy of Sciences, Moscow, Russia; ^8^Division of Infectious Diseases, Department of Medicine, Brigham and Women’s Hospital, Harvard Medical School, Boston, MA, USA; ^9^Belozersky Institute of Physico-Chemical Biology, Lomonosov Moscow State University, Moscow, Russia

**Keywords:** Gram-negative bacteria, lipid A, acyl chains, innate immunity, macrophages, proinflammatory cytokines

## Abstract

Toll-like receptor 4 (TLR4) is required for activation of innate immunity upon recognition of lipopolysaccharide (LPS) of Gram-negative bacteria. The ability of TLR4 to respond to a particular LPS species is important since insufficient activation may not prevent bacterial growth while excessive immune reaction may lead to immunopathology associated with sepsis. Here, we investigated the biological activity of LPS from *Burkholderia mallei* that causes glanders, and from the two well-known opportunistic pathogens *Acinetobacter baumannii* and *Pseudomonas aeruginosa* (causative agents of nosocomial infections). For each bacterial strain, R-form LPS preparations were purified by hydrophobic chromatography and the chemical structure of lipid A, an LPS structural component, was elucidated by HR-MALDI-TOF mass spectrometry. The biological activity of LPS samples was evaluated by their ability to induce production of proinflammatory cytokines, such as IL-6 and TNF, by bone marrow-derived macrophages. Our results demonstrate direct correlation between the biological activity of LPS from these pathogenic bacteria and the extent of their lipid A acylation.

## Introduction

Macrophages respond to pathogens by producing proinflammatory cytokines and reactive oxygen species (1). These mediators are involved in inflammatory and acute phase responses and have been implicated in host defense against pathogenic bacteria and parasites (2). The ability of the immune system to recognize pathogens relies on the expression of innate immune receptors by macrophages and other cell types (3). Toll-like receptor 4 (TLR4), the first specific receptor discovered for the mammalian innate immune response (4, 5), is capable of triggering a number of intracellular signaling pathways (6) leading to activation of a transcriptional program that leads to synthesis of proinflammatory cytokines (7).

TLR4 activation by its most prominent physiological ligand, lipopolysaccharide (LPS) (5) involves the formation of a complex with MD-2 protein and lipid A, which is the core structural component of LPS (8). Although the crystal structure of the LPS-MD-2/TLR4 complex has been determined (9), it is not entirely clear how structural variations in lipid A affect the ability of LPS from different bacteria to activate innate immunity through TLR4 (10, 11). A hexaacylated lipid A characteristic of *Escherichia coli*-type LPS is an agonist in all mammalian species tested, whereas its tetraacylated precursor called lipid IVa acts as an agonist in murine cells but is inactive for human macrophages and may even antagonize the action of potent agonists (12, 13). These lipid A variants are the most studied ligands of TLR4; however, the natural repertoire of LPSs is definitely much more diverse, in particular, with respect to the number and the length of fatty acid residues.

*Burkholderia mallei* is a Gram-negative bacterium that causes glanders, an infectious disease of horses that can be transmitted to humans by direct contact with infected animal or via food and water contamination (14, 15). Symptoms of acute infection with *B. mallei* in humans are pustular skin lesions and necrosis of the tracheobronchial tree following inhalation of the pathogens, or multiple abscesses and sepsis, if the skin is the site of entry (16). Interestingly, *B. mallei* was used as a biological weapon in time of the American Civil War and World Wars I and II (17).

*Pseudomonas aeruginosa* is one of the most frequent causes of nosocomial infections (18). Nosocomial pneumonia with multidrug-resistant strains of *P. aeruginosa* is a serious healthcare issue (19), especially in the developing countries (20). Interestingly, LPS from many *P. aeruginosa* strains isolated from patients with cystic fibrosis lacked the O-antigen, which resulted in higher sensitivity of the microorganism to bactericidal effects of the complement in normal human serum, yet this change correlated with chronic lung infections in cystic fibrosis patients (21).

The hospital environment is a reservoir for *Acinetobacter baumannii* (22). This agent can remain alive in the environment for prolonged periods of time due to innate resistance of its cells to desiccation (23). The proportion of pneumonia cases associated with *Acinetobacter* genus had increased from 4 to 7% through 1986–2003 (18). Moreover, *A. baumannii* can develop resistance to most frequently used antimicrobial agents, and the mortality associated with such highly resistant strains has been reported to reach as high as 40% (24, 25).

In this study, we investigated the biological activity of LPS variants isolated from *B. mallei*, *A. baumannii*, and *P. aeruginosa*, with a focus on their lipid A acylation status.

## Materials and Methods

### Bacterial Cultures and Isolation of LPS

The bacterial strains used in these studies, *E. coli* O130 (26), *Francisella tularensis* 15 (27), *A. baumannii* 1053 (28), *P. aeruginosa* 2192 (a clinical isolate from a cystic fibrosis patient) (29), and *B. mallei* C-5 (30), were grown as previously described.

The bacterial biomass was dried using acetone according to the standard protocol (31) or centrifuged, then frozen at −70°C and lyophilized.

Lipopolysaccharide samples from *E. coli* O130, *F. tularensis* 15, *B. mallei* C-5, and *A. baumannii* 1053 were isolated by the phenol–water extraction, as described (32). An LPS sample from *P. aeruginosa* 2192 was isolated by extraction with a mixture of aqueous 90% phenol/chloroform/light petroleum ether, as described (29). Highly purified preparations of LPS with a short-chain polysaccharide (R-form LPS) were obtained by gel chromatography on a column (35 cm × 2.5 cm) of AcA 44 Ultrogel in Tris-buffer (0.1M NaCl, 10 mM Tris, 1 mM EDTA, 0.25% Na-DOC) using UV detection at 206 nm the LPS-containing fractions were pooled and dialyzed first against 0.2% NaHCO_3_, then against distilled water and lyophilized. The purified LPS preparations were free from proteins and nucleic acids.

### Mass Spectrometry of LPS

MALDI-TOF mass spectrometry of purified LPS samples was performed on a Voyager STR system (PerSeptive, Framingham, MA, USA) and a 4800 Proteomic Analyzer (ABSciex, USA), as described (33). Negative ion mass spectra were acquired in both linear and reflector modes with mass accuracy ca. 50 ppm. 2′,4′,6′-Trihydroxyacetophenone monohydrate was used for matrix preparation. Mass spectra were analyzed as described (33).

### Laboratory Animals

C57Bl/6 mice and *Tlr4*-deficient mice were used at the age of 8–10 weeks (weight of 20–25 g). Mice were housed in the Pushchino Animal Breeding Facility (branch of the Shemyakin and Ovchinnikov Institute of Bioorganic Chemistry, Russian Academy of Sciences), under specific pathogen free conditions on 12-h light/dark cycle at room temperature. All manipulations with animals were carried out in accordance with recommendations in the Guide for the Care and Use of Laboratory Animals (NRC 2011), the European Convention for the Protection of Vertebrate Animals Used for Experimental and Other Scientific Purposes, Council of Europe (ETS 123), and “The Guidelines for Manipulations with Experimental Animals” (the decree of the Presidium of the Russian Academy of Sciences of April 02, 1980, no. 12000-496). All animal procedures were approved by Scientific Council of the Engelhard Institute of Molecular Biology.

### Cultivation and *In Vitro* Activation of Bone Marrow-Derived Macrophages

Murine bone marrow-derived macrophages (BMDM) were isolated from femurs and cultivated for 10 days according to the standard protocol (34) in DMEM (Gibco, USA) supplemented with 30% conditioned medium from L929 cells (a source of M-CSF) and 20% horse serum (HyClone, USA). To determine the mRNA levels of the cytokines, BMDM were plated on 12-well plates (10^6^ cells/ml) and stimulated with LPS for 2 h in a CO_2_-incubator at 37°C. To assess cytokines levels in the supernatants, BMDM were stimulated in 96-well plates (10^6^ cells/ml) for 4 h in a CO_2_-incubator at 37°C. After stimulation, the supernatant was transferred to fresh 96-well plate and stored at −80°C. LPS concentrations from 100 pg/ml to 100 ng/ml were tested in a series of preliminary experiments (Figure S1 in Supplementary Material). BMDM stimulation with 10 ng/ml of LPS consistently discriminated between different LPS species; therefore, that concentration was used in all subsequent experiments.

### Real-Time Quantitative RT-PCR Analysis

Total RNA from macrophages was isolated using the TRIzol Reagent (Invitrogen, Carlsbad, CA, USA) according to the manufacturer’s protocol. Reverse transcription was performed using 1.5 μg total RNA and random non-amers as primers with RevertAid First Strand cDNA Synthesis Kit (Thermo Scientific, USA) according to manufacturer’s protocol. Real-time quantitative PCR was performed using qPCRmix-HS SYBR + LowROX Kit (Evrogen, Moscow, Russia) on the ABI 7500 Real-Time PCR System (Applied Biosystems, Foster City, CA, USA). Amplifications were performed using the following program: preheating stage at 95°C for 10 min, 40 cycles at 95°C for 15 s, annealing at 61°C for 30 s, and extension at 72°C for 20 s. The following primers were used: IL-6, F: 5′-CTCTGCAAGAGACTTCCATCC, R: 5′-TTCTGCAAGTGCATCATCGT; TNF, F: 5′-TCTGTCTACTGAACTTCGGG, R: 5′-TTGGTGGTTTGCTACGAC; IL-1β, F: 5′-TCAACCAACAAGTGATATTCTCCAT, R: 5′-ACTCCACTTTGCTCTTGACTTCT; and β-actin, F: 5′-GACCTCTATGCCAACACAGT, R: 5′-AGAAAGGGTGTAAAACGCAG. Relative expression of target genes was determined using the ΔΔCt method and normalized to β-actin expression.

### ELISA Analysis

Murine IL-6 and TNF levels in cell-culture supernatants were measured using a Mouse IL-6 ELISA Ready-SET-Go! and Mouse TNFalpha ELISA Ready-SET-Go! kits (eBioscience, San Diego, CA, USA) according to the manufacturer’s protocol.

### Statistical Analysis

Statistical analysis was performed using GraphPad Prism software (version 6, San Diego, CA, USA). Two-tailed unpaired Student’s *t* tests were used for comparison of two independent data samples and determination of the degree of reliability. The data were obtained in at least three independent experiments and presented as the mean ± SEM. *P*-values <0.05 were considered to indicate statistical significance.

## Results

### Isolation and Mass Spectrometric Characterization of LPS

*Burkholderia mallei* possesses an S-form LPS consisting of lipid A, core oligosaccharide, and an O-specific polysaccharide chain (O-antigen) (35), whereas *A. baumannii* is characterized by a short-chain R-form LPS devoid of O-antigen (36). Wild-type strains of *P. aeruginosa* produce S-form LPS (37), but due to a mutation (38), cystic fibrosis isolates, including strain studied in this work (29, 38), are deficient in the O-antigen synthesis.

Lipopolysaccharides from *B. mallei* [a mixture of S- and R-forms (39)] and *A. baumannii* [a mixture of R-form and capsular polysaccharide (28)] were isolated by the phenol–water procedure (32). An attempt to extract R-form LPS from *P. aeruginosa* by this method failed, and therefore extraction with aqueous 90% phenol/chloroform/light petroleum ether was applied, which had been specially elaborated for this purpose (40). R-form LPS from each strain was purified by AcA 44 Ultrogel gel chromatography.

Structures of the lipid moiety of the LPS (lipid A) were determined by MALDI-TOF mass spectrometry in the negative ion mode taking into account reported basal structures of lipid A of *B. mallei* (39), *A. baumannii* (41), and *P. aeruginosa* (42, 43). Lipid A of all bacteria studied were found to have a bisphosphorylated glucosamine disaccharide backbone, which is most typical of Gram-negative bacteria (44). Distribution of fatty acids between the glucosamine residues was established by MS/MS analysis.

### Structure Determination of Lipid A of *A. baumannii*

**Figure 1:**
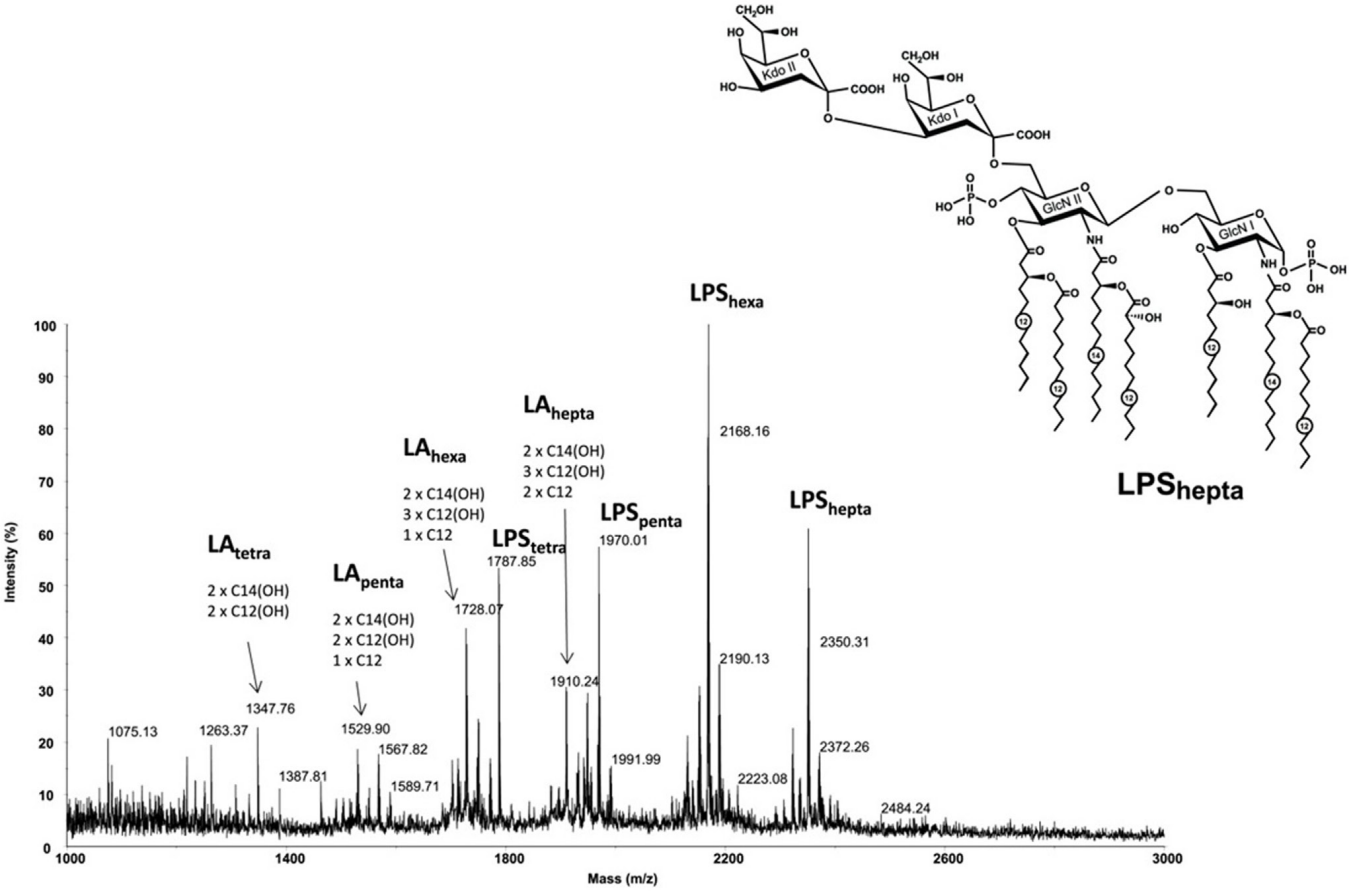
**High resolution MALDI-TOF mass spectrum of *Acinetobacter baumannii* 1053 LPS recorded in the negative ionization reflectron mode**. Structure of the heptaacyl lipid A in LPS from *A. baumannii*. Kdo, 3-deoxy-d-*manno*-oct-2-ulosonic acid; GlcN, glucosamine. Numbers indicate the number of carbon atoms in the acyl chain.

Lipid A of *A. baumannii* 1053 strain predominantly bears six residues of fatty acids along with some species containing seven, five, and even four fatty acid residues (Figure 1). In addition to the lipid A moiety, complete LPS molecule contains two 3-deoxy-d-*manno*-oct-2-ulosonic acid residues attached to the lipid A backbone (the most abundant species is LPS_hexa_, *M*_exp_ = 2168.16, *M*_calc_ = 2168.23). The peaks in the lower mass regions corresponded to lipid A species (LA_tetra_ to LA_hepta_) that originated from the in-source fragmentation of the LPS (33).

The distribution of the fatty acids in the lipid A from *A. baumannii* was established based on the MS/MS fragmentation of the LA_hepta_ ion at *m*/*z* 1910.24. Conceptually, it is a MS^3^ spectrum obtained with a MS/MS instrument since the parent ion is already a Y-type fragment originating from the in-source decay of the LPS quasi-molecular ion. The spectrum (not shown) contained a Y-fragment peak at *m*/*z* 864.7, which allowed identification of the fatty acids attached to glucosamine I and glucosamine II of the lipid A backbone. The spectrum also contained peaks at *m*/*z* 1710 and 1694, which corresponded to a loss of C12 and 3HOC12 residues, respectively. Overall, the predominant hexaacylated form of the lipid A contained four primary fatty acids (two N-linked 3HOC14 fatty acids and two O-linked 3HOC12 residues) and two secondary fatty acids (one 3HOC12 and one C12). The heptaacylated form of the lipid A contained an additional C12 residue and has the structure shown in Figure 1, whereas penta- and tetraacyl lipids A lacked one or two C12 or C12 and 3HOC12 residues. In addition to the presence of sodium adduct ions, the main sources of heterogeneity in the spectra were associated with replacement of one or two residues of 3HOC12 or C12 with 3HOC14 or C14.

### Structure Determination of Lipid A of *B. mallei*

**Figure 2:**
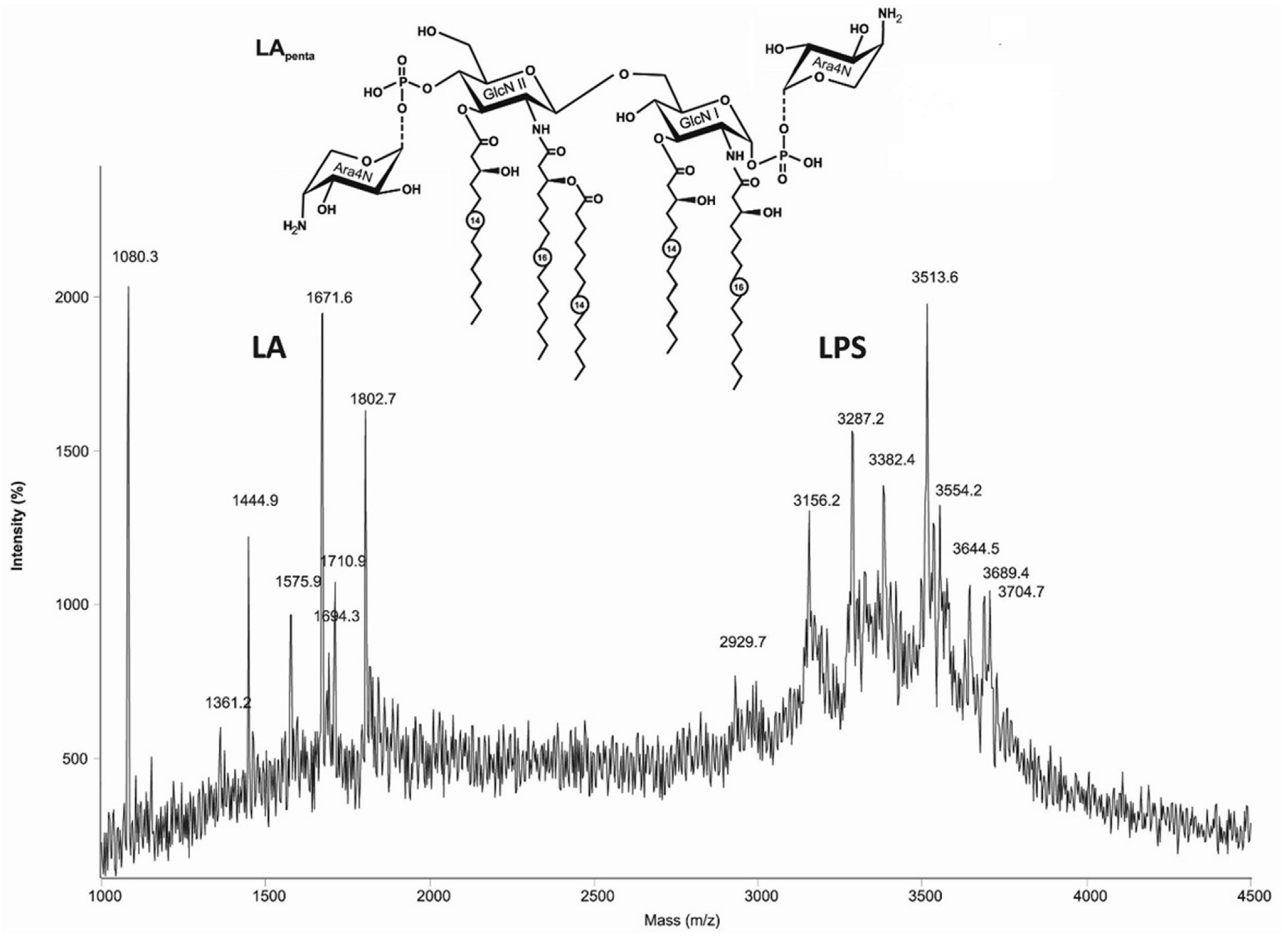
**The MALDI-TOF mass spectrum of the LPS from *Burkholderia mallei* C-5 contained two clusters of ions, corresponding to lipid A fragments and intact LPS molecules**. Structure of the pentaacyl lipid A in LPS from *B. mallei*. Ara4N, 4-amino-4-deoxyarabinose; GlcN, glucosamine. Numbers indicate the number of carbon atoms in the acyl chain. Dotted lines indicate non-stoichiometric substitution.

The MALDI-TOF mass spectrum of the LPS from *B. mallei* C-5 strain (Figure 2), acquired in linear mode, contained two clusters of ions between *m*/*z* ~1300 and 2000, corresponding to lipid A fragments and the core oligosaccharide at *m*/*z* 1710.9, as well as intact LPS molecules in the mass range at *m*/*z* 2900–4000 (Figure 2). Lipid A of *B. mallei* was characterized by longer chain fatty acids. Pentaacylated lipid A (*m*/*z* 1671.6) includes two N-linked residues of 3HOC16 and two *O*-linked residues of 3HOC14 as primary acyl groups and one secondary residue of C14 fatty acid. The tetraacylated form (*m*/*z* 1444.9) was present in smaller amounts and comprised one residue of 3HOC14. A characteristic feature of these LPS is the presence of significant amount of tetra- and pentaacylated lipid A (*m*/*z* 1575.9 and 1802.7, respectively), bearing one residue of cationic monosaccharide 4-amino-4-deoxyarabinose (Ara4N) attached to one of the phosphate groups giving the dominant quasi-molecular ions at *m*/*z* 3287.2 and 3513.6.

### Structure Determination of Lipid A of *P. aeruginosa*

**Figure 3:**
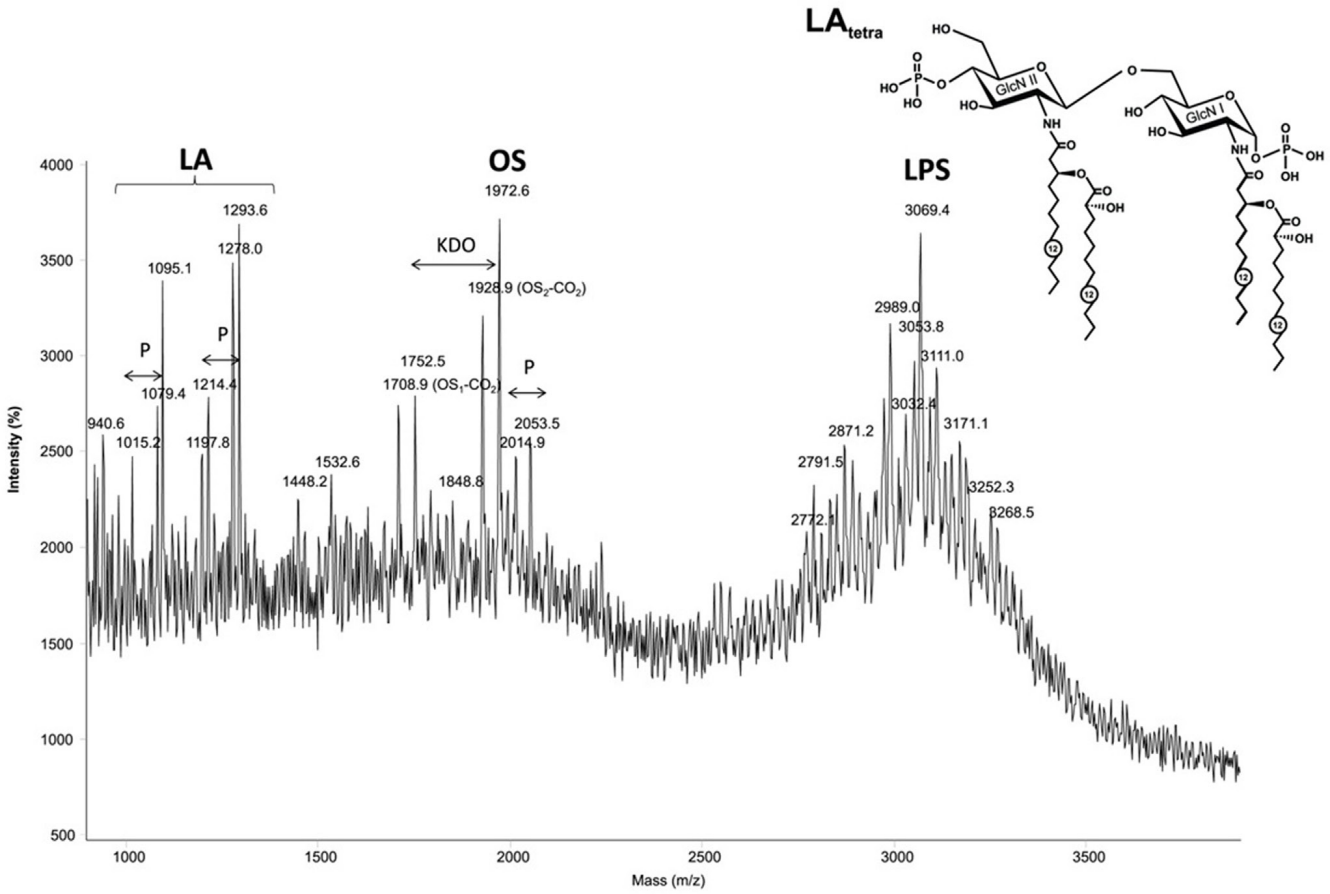
**The MALDI-TOF mass spectrum of the *Pseudomonas aeruginosa* 2192 in the linear negative mode contained three distinct clusters of ions, corresponding to lipid A fragments, core fragments and intact LPS, respectively (listed in the order of increasing molecular mass)**. Structure of the tetraacyl lipid A in LPS from *P. aeruginosa*. GlcN, glucosamine. Numbers indicate the number of carbon atoms in the acyl chain.

The MALDI-TOF mass spectrum of the *P. aeruginosa* 2192 LPS (Figure 3) obtained in the linear negative mode contained three distinct clusters of ions, corresponding to lipid A fragments, core fragments and intact LPS, respectively (listed in the order of increasing molecular mass). The MS/MS analysis of the oligosaccharide (spectrum not shown) revealed a molecular structure in agreement with the published *P. aeruginosa* core oligosaccharide (29). The lipid A portion of the LPS is the least acylated of all LPS studied in this work, with almost equal amounts of tri- and tetraacylated forms. The tetraacylated form included two primary and two secondary residues 3HOC12 (Figure 3), whereas the triacylated form contained only one secondary residue 3HOC12. The heterogeneity was associated with the absence in some of the molecules of one of two phosphate groups and partial replacement of one secondary fatty acid with its non-hydroxylated form (C12).

### The Length and the Number of Lipid A Acyl Chains Directly Correlates with the Biological Activity of LPS

The biological activity of LPS samples was assessed by their ability to induce production of proinflammatory cytokines by BMDM isolated from WT mice. For mRNA levels, cells were incubated with 10 ng/ml LPS for 2 h, and protein concentration in the culture medium was measured 4 h post LPS treatment. Highly active LPS from *E. coli* with hexaacyl biphosphoryl lipid A (45) and inactive LPS from *F. tularensis* with tetraacyl monophosphoryl lipid A (46) were used as positive and negative controls, respectively. Another negative control that was utilized and allowed us to assess the specificity of TLR4 signaling was the use of BMDM cultures prepared from *Tlr4*-deficient mice (Figure S2 in Supplementary Material). Both negative controls demonstrated the lack of measurable expression of proinflammatory cytokines.

**Figure 4:**
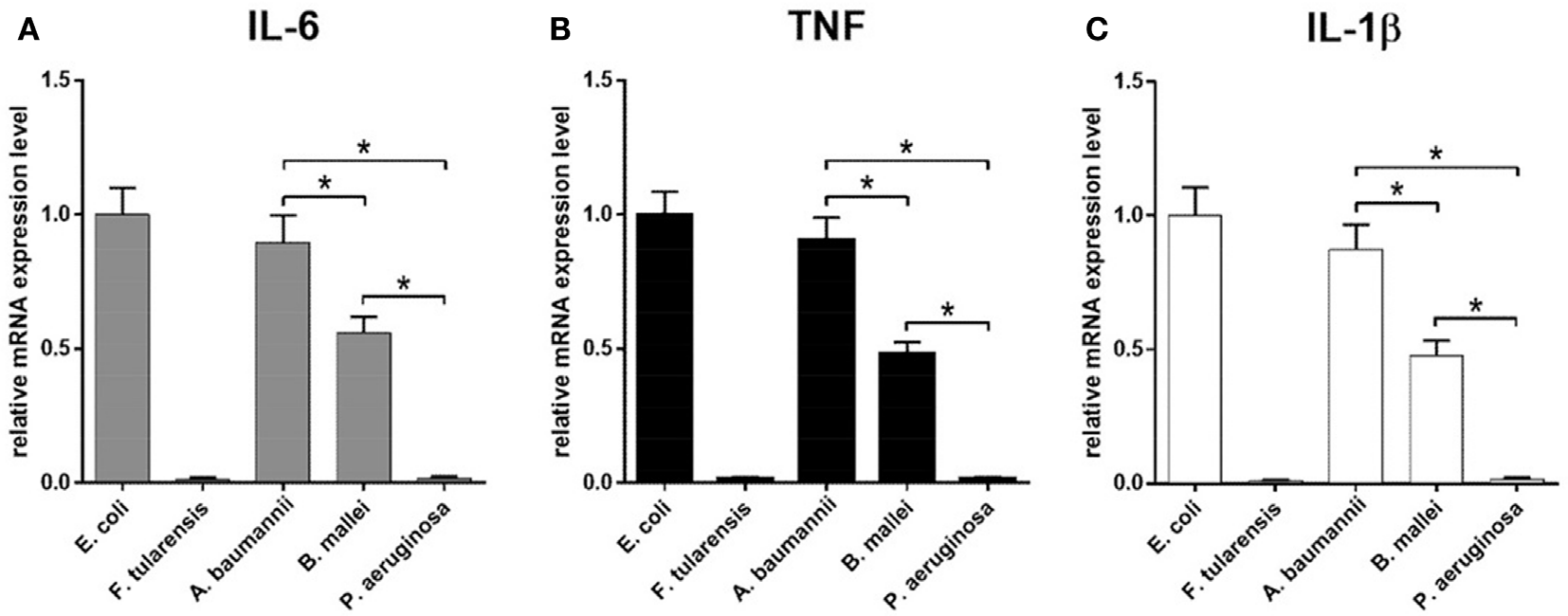
**Induction of mRNA of proinflammatory cytokines in BMDM activated by LPS isolated from various bacteria**. Quantification of IL-6 **(A)**, TNF **(B)**, and IL-1β **(C)** mRNA levels in BMDM isolated from WT mice. Relative mRNA expression levels were normalized to β-actin. All data are representative of three or more independent experiments. Data represent mean values ± SEM. *P < 0.05, as calculated by Student’s *t* test.

**Figure 5:**
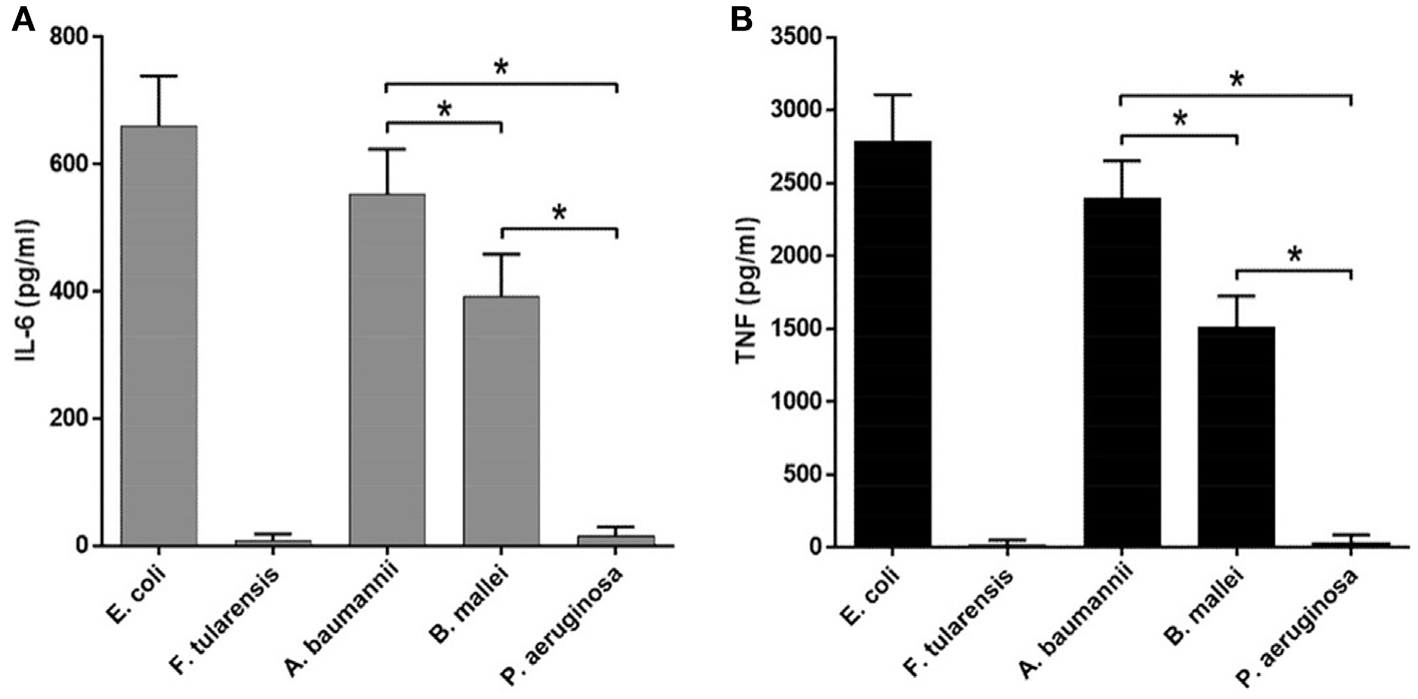
**Expression of proinflammatory cytokines by BMDM upon sactivation by LPS from various bacteria**. ELISA quantification of IL-6 **(A)** and TNF **(B)** level in the supernatants of LPS-stimulated BMDM of WT mice. All data are representative of three or more independent experiments. Data represent mean values ± SEM. *P < 0.05, as calculated by Student’s *t* test.

Similar results were observed in the experiments measuring the mRNA expression and cytokine production, indicating that all effects were primarily transcriptional, consistent with direct effects of the lipid A structure on the strength of TLR4 activation (Figures 4 and 5). In addition, all proinflammatory cytokines measured demonstrated virtually identical expression profiles with regard to LPS source, at both mRNA (IL-6, TNF, and IL-1β, Figures 4A–C) and protein (IL-6 and TNF, Figures 5A,B) levels.

Lipopolysaccharide isolated from *A. baumannii* demonstrated the biological activity similar to that of LPS isolated from *E. coli* (Figures 4 and 5), consistent with the presence of highly acylated lipid A in both strains. Interestingly, the former was a slightly weaker activator than the latter, even though the difference did not reach the level of statistical significance. This trend can be explained by the difference between the average length of acyl groups in the most abundant hexa- and heptaacylated forms of *A. baumannii* lipid A compared to *E. coli* lipid A. In *A. baumannii* lipid A, most of the acyl groups have 12 carbon atoms and only one or two have 14 carbon atoms, whereas in *E. coli* lipid A most of the acyl chains are C14 derivatives.

Lipid A of *B. mallei* had, on the average, longer acyl chains (C14–16) as compared to the lipid A of *E. coli* and displayed significantly lower biological activity (Figures 4 and 5), in agreement with the lower degree of acylation: the major forms of *B. mallei* lipid A were penta- and tetraacylated, whereas lipid A from *E. coli* was hexaacylated.

Lipopolysaccharide isolated from *P. aeruginosa* induced virtually no expression of cytokines by BMDM (Figures 4 and 5). The apparent lack of biological activity of this LPS correlated well with the low acylation of its lipid A, which consisted mainly of tri- and tetraacylated forms. In addition, ~30% of the *P. aeruginosa* lipid A molecules lacked one of the phosphate groups and the length of the acyl chains was on the average shorter (C12) than that in lipid A of *A. baumannii* (C12–14) and *B. mallei* (C14–16).

## Discussion

In the present study, we assessed the biological activity of LPS from the pathogenic bacteria *B. mallei*, *A. baumannii*, and *P. aeruginosa* in order to establish the structure–function correlations for LPS species from various Gram-negative bacteria, namely, the length and the number of the acyl chains in lipid A, and its biological activity as a TLR4 agonist. Previously, we compared the LPSs from ancient psychrotrophic bacteria of the genus *Psychrobacter* to those from wild-type and mutant strains of *Yersinia pestis* (47). The decrease in the chain length to C10–12 in the LPS of *Psychrobacter* spp. (as compared to C14 in the highly active LPS of *E. coli*) resulted in a significant drop of its biological activity. A similar trend was observed for LPS from *A. baumannii* (Figures 4 and 5), indicating that lipid A with longer carbon atom acyl chains in fatty acid residues is a stronger cytokine inducer. At the same time, lipid A acyl chains in *B. mallei* were on the average longer (C14–16) than those in *E. coli* (C14), yet LPS from *B. mallei* appeared to be a weaker activator. However, lipid A from *B. mallei* is also less acylated and contains cationic monosaccharides, similar to the lipid A of *Burkholderia cenocepacia* (48). Indeed, lipid A from *B. mallei* contains Ara4N residue in almost half of the molecules, which would partially neutralize the negative charge of the phosphate groups necessary for the interaction with the positively charged amino acids of TLR4 (11). Consistent with our data, it has been recently demonstrated that in the case of low-acylated lipid A of *B. cenocepacia*, the length of the acyl chains and the presence of Ara4N significantly affected the biological activity of LPS (10).

Lipopolysaccharide species from *P. aeruginosa* was inactive in our assays, inducing no measurable cytokine production by BMDM (Figures 4 and 5). Interestingly, lipid A from *P. aeruginosa* strain studied in this work contains a maximum of four acyl chains and therefore appears underacylated as compared to published data on the degree of *P. aeruginosa* lipid A acylation, which varies from strain to strain and depends on the environmental conditions (42, 43). Nevertheless, lipid IVa from *E. coli* (49) and LPS from *Y. pestis* ΔlpxM/ΔlpxP mutant (47) which contain tetraacylated lipid A can induce the production of cytokines by murine macrophages, albeit at lower levels. This observation suggests that tetraacylated lipid A is minimally required yet not sufficient to fully engage TLR4 in signal transduction. The difference between active and inactive tetraacylated lipid A may be attributable to the total charge of this molecule defined by the presence or absence of negatively charged phosphate groups and positively charged Ara4N residues (50) as well as to a different fatty acid chain length (C12 in the inactive *P. aeruginosa* tetraacylated lipid A versus C14 in the active *E. coli* lipid IVa).

Structure–function relationships for distinct LPS species are clinically important for a number of reasons. Pathogenic bacteria may employ LPS with low biological activity to evade proper recognition by the TLR4/MD-2 system, dampening the host immune response and increasing the risk of bacterial dissemination. On the other hand, such LPS would not be able to induce septic shock in susceptible patients (51), rendering septic complications more manageable. Yet, defining and understanding how even the smallest structural differences between the very similar bacterial ligands may affect the activation of the immune response may provide the mechanism for the fine tuning of the latter and new insights to immunomodulatory processes.

In summary, our results demonstrate that the ability of LPS to activate production of the key proinflammatory cytokines, in particular IL-6, TNF, and IL-1β, by engaging TLR4 on murine macrophages steadily decreases with the number and the length of the lipid A acyl chains. We also observed that the number of acyl groups in lipid A affected LPS activity more potently than their length. Further studies are needed to define relative contributions of other lipid A structural features to the biological activity of LPS.

## Author Contributions

KK, MD, YK, and DK analyzed literature and designed research. KK, NA, AP, RS, MS, ANK, ES, and AAK performed experiments. KK, AM, GP, LS, DG, SN, YK, and DK wrote the manuscript. All authors analyzed data and revised the manuscript.

## Conflict of Interest Statement

The authors declare that the research was conducted in the absence of any commercial or financial relationships that could be construed as a potential conflict of interest.

## Abbreviations

Ara4N, 4-amino-4-deoxyarabinose; BMDM, bone marrow-derived macrophages; IL, interleukin; LPS, lipopolysaccharide; MALDI-TOF MS, matrix-assisted laser desorption/ionization time-of-flight mass spectrometry; M-CSF, macrophage colony-stimulating factor; MD-2, myeloid differentiation factor 2; TLR, toll-like receptor; TNF, tumor necrosis factor.
