# Supplementary material for: Structural Relationship of the Lipid A Acyl Groups to Activation of Murine Toll-Like Receptor 4 by Lipopolysaccharides from Pathogenic Strains of Burkholderia mallei, Acinetobacter baumannii, and Pseudomonas aeruginosa
Source: Front Immunol. 2015 Nov 23;6:595. doi: 10.3389/fimmu.2015.00595 (PMC4655328; doi:10.3389/fimmu.2015.00595)
Supplement: Supplementary file 1 [file presentation_1.ppt]

## Slide 1
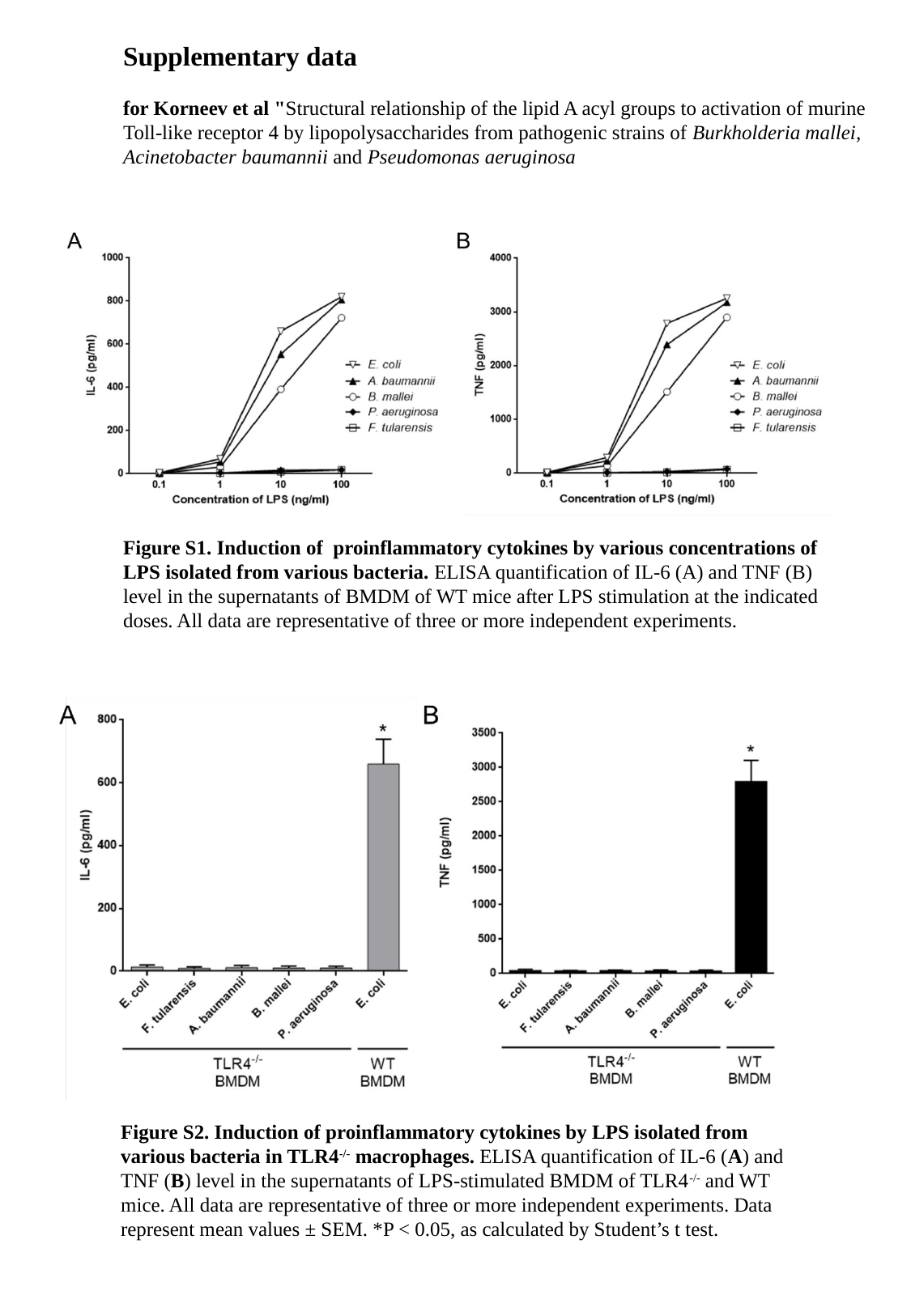

Supplementary data
for Korneev et al "Structural relationship of the lipid A acyl groups to activation of murine Toll-like receptor 4 by lipopolysaccharides from pathogenic strains of Burkholderia mallei, Acinetobacter baumannii and Pseudomonas aeruginosa
Figure S1. Induction of proinflammatory cytokines by various concentrations of LPS isolated from various bacteria. ELISA quantification of IL-6 (A) and TNF (B) level in the supernatants of BMDM of WT mice after LPS stimulation at the indicated doses. All data are representative of three or more independent experiments.
Figure S2. Induction of proinflammatory cytokines by LPS isolated from various bacteria in TLR4-/- macrophages. ELISA quantification of IL-6 (A) and TNF (B) level in the supernatants of LPS-stimulated BMDM of TLR4-/- and WT mice. All data are representative of three or more independent experiments. Data represent mean values ± SEM. *P < 0.05, as calculated by Student’s t test.
